# Neuroscience of Compulsive Eating Behavior

**DOI:** 10.3389/fnins.2017.00469

**Published:** 2017-08-24

**Authors:** Catherine F. Moore, Valentina Sabino, George F. Koob, Pietro Cottone

**Affiliations:** ^1^Laboratory of Addictive Disorders, Departments of Pharmacology and Psychiatry, Boston University School of Medicine Boston, MA, United States; ^2^Graduate Program for Neuroscience, Boston University School of Medicine Boston, MA, United States; ^3^National Institute on Alcohol Abuse and Alcoholism, National Institutes of Health Bethesda, MD, United States

**Keywords:** compulsive, addiction, eating, habit, withdrawal, inhibitory control

## Abstract

A systematic characterization of compulsivity in pathological forms of eating has been proposed in the context of three functional domains: (1) habitual overeating; (2) overeating to relieve a negative emotional state; and (3) overeating despite aversive consequences. In this review, we provide evidence supporting this hypothesis and we differentiate the nascent field of neurocircuits and neurochemical mediators of compulsive eating through their underlying neuropsychobiological processes. A better understanding of the neurobiological mechanisms that lead to compulsive eating behavior can improve behavioral and pharmacological intervention for disorders of pathological eating.

## Introduction

Compulsivity, defined as a strong, irresistible internal drive to perform an action, typically contrary to one's will, is a transdiagnostic construct present in numerous psychiatric conditions. Compulsive eating behavior is observed in pathological forms of feeding behavior, such as binge eating disorder (BED), certain forms of obesity, and the recently proposed “food addiction” (Moore et al., 2017). BED is an eating disorder defined by uncontrolled overeating of palatable food (i.e., high in fat and/or sugar) in brief periods of time. “Food addiction” is a recently proposed concept measured by the “Yale Food Addiction Scale,” which uses diagnostic criteria based on the Diagnostic and Statistical Manual (DSM-V) diagnosis of substance use disorder (American Psychiatric Association, 2013; Gearhardt et al., 2016). While compulsive eating behavior is highly prevalent in obese individuals, it is neither necessary nor sufficient to characterize obesity, an extremely heterogeneous disorder defined simply through having a body mass index (BMI) of ≥30 (Curtis and Davis, 2014). Here, we review evidence from the literature supporting the dissection of compulsive eating into three main elements: (1) habitual overeating (Smith and Robbins, 2013; Tomasi and Volkow, 2013), (2) overeating to relieve a negative emotional state (Cottone et al., 2009a; Parylak et al., 2011), and (3) overeating despite aversive consequences (Cottone et al., 2012; Rossetti et al., 2014). In animal models, long-term access to palatable food results in compulsive-like habit formation, which results in negative emotion-like states and which is resistant to aversive consequences. It is important to note that the different elements of compulsive eating are not mutually exclusive, and can be attributed to distinct, though often intersecting, mechanisms.

## Habitual overeating

Goal-directed, voluntary actions can become compulsive, stimulus-driven habits through Pavlovian conditioning mechanisms. Habits are formed when the stimulus-response association overlaps the goal of the behavior (e.g., the palatable food or the drug); and the outcome/reward no longer motivates the action (Everitt and Robbins, 2016). This overlap occurs throughout repeated pairings, where reward-associated stimuli (e.g., advertising in humans, or a tone in animals) can elicit and maintain compulsive seeking behavior (Everitt and Robbins, 2016). An important element of compulsive habits is the inability to retain evaluative processes that can allow for the switch from *stimulus-response* driven back to *goal-directed* actions when the value of the reward is reduced (Watson et al., 2014; Horstmann et al., 2015). Therefore, habitual behavior can be assessed through outcome devaluation procedures, where persistence of responding is measured after the value of the outcome is decreased (i.e., drug/food reward).

**Figure 1:**
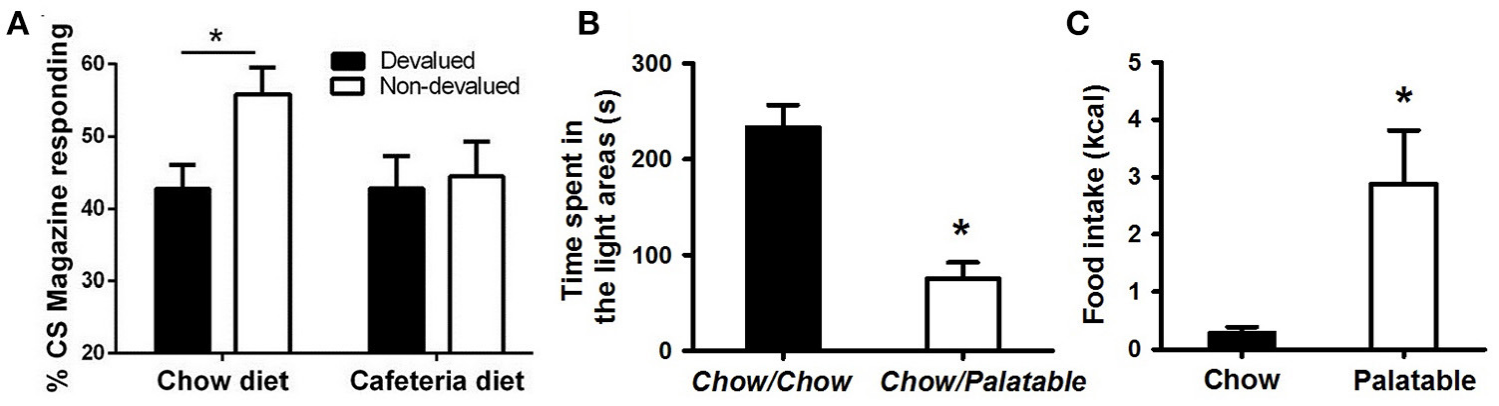
Evidence of compulsive eating behavior in animal models. **(A)** Rats fed a cafeteria diet show resistance to devaluation procedures; from Reichelt et al. (2014). In this procedure, rats were fed continuously a high-fat cafeteria diet, consisting of four commercially available foods: half-sweet (cakes, cookies) and half-savory (pies, dim sims) items. After 2 weeks, rats were trained to consume sucrose deliveries (either cherry or grape flavored) paired with a stimulus (tone or noise, respectively). Following training, one of these sucrose solutions was devalued by specific satiety, where rats could drink one particular flavor *ad libitum* prior to testing. Head entries during sucrose-paired stimulation were measured for both the devalued and non-devalued flavor of sucrose. A decrease in head entries compared to the non-devalued condition indicates devaluation, or goal-directed behavior; whereas no decrease in head entries indicates habitual behavior. **(B)** Rats withdrawn from a highly palatable, chocolate diet display anxiety-like behaviors; adapted with permission from Iemolo et al. (2013). In this paradigm, animals are either continuously fed a standard chow food (“Chow/Chow”) or intermittently cycled between a standard chow food for 5 days and a highly-palatable, high-sucrose food for 2 days (“Chow/Palatable”). After chronic, intermittent palatable diet exposure rats were tested for anxiety-like behavior in a light/dark apparatus during withdrawal from chronic, intermittent access to the palatable diet. Shorter time spent in the light side of the apparatus indicates higher anxiety-like behavior compared to “Chow/Chow” control rats. **(C)** Food intake in rats with a history of intermittent access to palatable food is resistant to the aversiveness of a light/dark conflict test; adapted with permission from Ferragud et al. (2017). In this experiment, animals were trained in an operant chamber to self-administer food pellets that consisted of a standard chow food (“Chow”) or a highly-palatable, high-sucrose food (“Palatable”) for 1 h each day. Following escalation of palatable food responding, animals were tested for compulsive-like behavior in the light/dark conflict test. This test consists of a light/dark apparatus where a food cup containing the same food received during self-administration is positioned in the aversive, light compartment. “Compulsive-like eating” is operationalized as the amount of food eaten during the trial compared to control “Chow” conditions, where eating behavior is typically suppressed due to the aversiveness of the light compartment. ^*^p < 0.05 Bonferonni corrected.

There is evidence from both human and animal studies to suggest a link between binge eating/palatable food and an increased tendency to engage in habitual responding. Individuals with BED and/or obesity have been shown to display a bias toward habitual responding (Horstmann et al., 2015; Janssen et al., 2017) and the use of neural circuits that support these processes (Voon et al., 2015). In addition, palatable food consumption induced habitual responding in animals, observed as resistance to devaluation procedures (Kendig et al., 2013; Furlong et al., 2014; Reichelt et al., 2014; Figure 1A). Furthermore, in healthy weight controls, palatable food associated cues bias responding away from goal-directed actions toward habitual behavior, determined as continued cue-elicited food seeking after satiation (i.e., resistance to devaluation; Watson et al., 2014). Obese and binge eating individuals showed heightened food cue reactivity and attentional biases (Carnell et al., 2014; Schmitz et al., 2014), which likely contribute to the initiation and the persistence of overeating.

**Figure 2:**
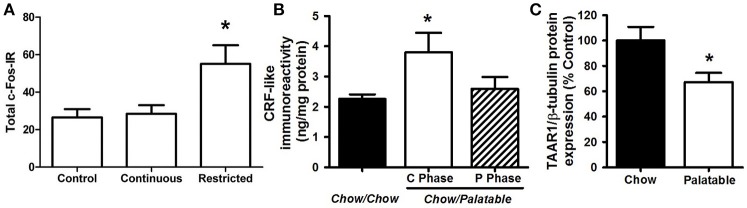
Neurobiological substrates of compulsive eating behavior in animal models. **(A)** Rats that habitually overeat display increased activation of the dorsolateral striatum; from Furlong et al. (2014). In this procedure, rats were given either control chow only, or chow + continuous or restricted (2 h daily) access to palatable food, consisting of sweetened-condensed milk (22% fat; 67% sugar, 10% protein). After 2 weeks, rats were tested in a devaluation procedure to assess habitual responding for food reward. The restricted access group displayed resistance to devaluation procedures, or habitual responding for palatable food, while control animals and the continuous access group retained goal-directed responding. Following this procedure, cFos immunoreactivity (cFos-IR) was quantified in the dorsolateral striatum, and habitually responding, restricted access rats displayed an increase in cFos IR compared to control and continuous access groups. **(B)** Rats withdrawn from a highly palatable, chocolate diet display increased CRF in the CeA; adapted with permission from Cottone et al. (2009a). In this paradigm, animals are either continuously fed a standard chow food (“Chow/Chow”) or intermittently cycled between a standard chow food for 5 days and a highly-palatable, high-sucrose food for 2 days (“Chow/Palatable”). After chronic, intermittent palatable diet exposure rats display anxiety- and depressive-like behavior during withdrawal from palatable food. This negative emotional state is accompanied by an increase in CRF expression in the CeA of withdrawn rats. **(C)** Compulsive, binge eating rats have reduced TAAR1 expression in the PFC; adapted with permission from Ferragud et al. (2017). In this experiment, animals were trained in an operant chamber to self-administer food pellets that consisted of a standard chow food (“Chow”) or a highly-palatable, high-sucrose food (“Palatable”) for 1 h each day. Following escalation of palatable food responding, “Palatable” rats display compulsive-like eating in the light/dark conflict test, as well as a decrease in TAAR1 expression in the PFC. ^*^p < 0.05.

The transition from reinforcement learning to habitual responding is hypothesized to be mediated by the striatum, an area composed of ventral (i.e., nucleus accumbens, NAc) and dorsal regions. While the NAc plays a key role in the reinforcing effects of food and drugs, the dorsal striatum is thought to contribute to the development of habits (Everitt and Robbins, 2016). Habit-learning processes, implicated in the shift to addiction, are accompanied by a concomitant shift from ventral to dorsal striatal circuits that control behavior. Food and associated cues increase extracellular dopamine transmission in the NAc, which is hypothesized to result in increased incentive salience and an enhancement of habit learning (Everitt and Robbins, 2016). Initially in reinforcement learning, which corresponds with early stages of drug use or palatable food consumption, dopamine signaling in the NAc drives goal-directed responding for the reward, and the pharmacological inactivation of the dorsal striatum has no effect. However, in later stages, when habitual responding eventually dominates, antagonizing dorsolateral striatal dopamine blocks compulsive-like responding and restores sensitivity to devaluation (Belin and Everitt, 2008). Research indicates heightened behavioral and/or neural responses to food cues in individuals with BED (Wang et al., 2011) and obesity (Stoeckel et al., 2008; Carnell et al., 2014), and behavioral and/or neural responses to food cues can predict subsequent food intake and weight gain (Demos et al., 2012; Lawrence et al., 2012). Dopamine-2 receptor (D2R) binding potential in dorsal regions of the striatum was found to be positively associated with BMI and habitual, opportunistic eating (Guo et al., 2014) yet D2R availability in the entire striatum has also been found to be lower in obese individuals (further discussed below; Volkow et al., 2008), likely reflecting dynamic and regional changes as habitual compulsive overeating evolves. For example, one interpretation of the decreased D2R availability is that dopamine function becomes compromised with repeated excessive activation, see below. This highlights the importance of researchers further associating neurobiological measures with behavioral indices of habitual compulsive overeating. In an animal model, long-term, intermittent access to palatable food was associated with greater activation of the dorsolateral striatum in rats (Figure 2A; Furlong et al., 2014). Thus, experience with palatable food causes neuroadaptations in striatal circuitry, which may, in turn, cause and potentiate compulsive, habitual overeating, and increase susceptibility to food cues.

## Overeating to relieve a negative emotional state

In drug addiction, the transition from casual to compulsive drug use is hypothesized to reflect an allostatic change in mood, where drugs acquire negative reinforcing properties (Koob et al., 2014). Analogously, ingesting palatable food to alleviate a negative emotional state represents an element of compulsive eating behavior (Cottone et al., 2009a; Parylak et al., 2011). Two neuropsychobiological processes underlie this element: *(i)* decreased reward function, caused by within-system neuroadaptations and *(ii)* withdrawal-induced negative affect, caused by between-system neuroadaptations (Parylak et al., 2011; Koob et al., 2014). These processes are characterized by affective habituation and loss of motivation for ordinary life stimuli, as well as by dysphoria, irritability, and anxiety (Parylak et al., 2011; Koob et al., 2014). Through a negatively reinforced mechanism, compulsive eating would, therefore, “paradoxically” both improve the reward deficit and suppress negative emotions in the short term, but worsen them in the long term, a form of misregulation in self-regulation theory (Koob and Le Moal, 1997; Cottone et al., 2009a; Parylak et al., 2011).

The overeating of, and subsequent withdrawal from, palatable food is hypothesized to cause or contribute to the negative emotional state. In humans, there is evidence that dieting contributes to negative affect, which in turn predicts later increases in eating pathology (Stice, 2002). Indeed, consumption of palatable food, commonly referred to as “comfort food,” can effectively mitigate acutely the physiological stress response and anxiety (Pecoraro et al., 2004; Tomiyama et al., 2011), thus compulsive eating behavior may be strengthened through negative reinforcing mechanisms. Similarly, in animal models, overconsumption of palatable diets has been shown to decrease brain reward system functioning; for example, decreases in brain-stimulation reward responsiveness in obese rats (Johnson and Kenny, 2010) and decreased motivation for rewards in animals with a history of prolonged palatable food consumption (Vendruscolo et al., 2010). During withdrawal from palatable food, the emergence of a negative emotional state, characterized by anxiety- and depressive-like behavior (Figure 1B; Iemolo et al., 2012) (Cottone et al., 2008; Sharma et al., 2013), and enhanced stress-responsiveness (Avena et al., 2008; Blasio et al., 2014a), is observed. When access to palatable food is renewed, subjects display compulsive-like eating (Avena et al., 2008; Cottone et al., 2009b; Rossetti et al., 2014) and withdrawal-induced anxiety- and depressive-like behaviors are paradoxically reversed (Iemolo et al., 2012). Thus, evidence suggests the emergence of a negative emotional state is induced by withdrawal from palatable food, and that compulsive eating behavior is driven by its ability to relieve such a state.

Within-system neuroadaptations are hypothesized to occur during palatable food overconsumption, which may repeatedly stimulate and eventually desensitize the mesolimbic system, resulting in reward deficiency. There is evidence of reduced dopaminergic signaling in striatal regions of obese individuals, observed as lower D2R availability (Volkow et al., 2008), reduced neural response to consummatory food reward (Stice et al., 2008, 2010), and blunted amphetamine-induced dopamine release in the NAc (van de Giessen et al., 2014). High fat and high sugar diet exposure has been shown to alter dopaminergic signaling observed as downregulation of striatal D2Rs (Johnson and Kenny, 2010), decreases in baseline extracellular dopamine in the NAc (Zhang et al., 2015), and decreases in dopamine transporter expression and function (Hajnal and Norgren, 2002; Hryhorczuk et al., 2016). Thus, it is hypothesized that palatable food-related reward is diminished, and compulsive overeating reflects an attempt to reactivate a hypofunctional reward circuit (Wang et al., 2001; Geiger et al., 2009).

Between-system neuroadaptations are also hypothesized to cause the emergence of a negative emotional withdrawal state and are largely characterized by recruitment of stress systems in the extended amygdala (Koob et al., 2014), an area consisting of the central nucleus of the amygdala (CeA), the bed nucleus of the stria terminalis (BNST), and a transition area in the NAc shell. In the CeA, corticotropin-releasing factor (CRF) and its type-1 receptor (CRF1R) are recruited following extended access to palatable food (e.g., palatable food withdrawal-induced increases in CRF expression and CRF1R electrophysiological responsiveness) (Figure 2B; Cottone et al., 2009a) (Teegarden and Bale, 2007; Iemolo et al., 2013). Increased anxiety-like behavior observed during withdrawal is mediated by the CRF-CRF1 system in the CeA (Cottone et al., 2009a; Iemolo et al., 2013), and renewed consumption of the palatable diet reverses both the withdrawal-dependent behaviors and the heightened CRF expression levels (Teegarden and Bale, 2007; Cottone et al., 2009a; Iemolo et al., 2013). In addition, stress-induced overconsumption of the palatable diet can be blocked by administration of CRF1R antagonists into the BNST (Micioni Di Bonaventura et al., 2014). These studies indicate a critical role for CRF-CRF1R system in both food withdrawal-like behavior and negative reinforcement-driven palatable food overconsumption.

During withdrawal, the endocannabinoid system is engaged, likely to compensate for the recruitment of the CRF-system, and acts to restore homeostasis in amygdalar circuits (Sidhpura and Parsons, 2011; Koob et al., 2014). The endocannabinoid system of the amygdala is hypothesized to serve as a “buffer system” to dampen the negative emotional state driven by withdrawal with food and drugs (Sidhpura and Parsons, 2011; Blasio et al., 2013; Koob et al., 2014). Indeed, withdrawal from palatable food recruited the endocannabinoid system in the CeA, induced the upregulation of 2-arachidonoylglycerol (2-AG) and cannabinoid type-1 receptor (CB1R) (Blasio et al., 2013). Blocking CB1R with the inverse agonist rimonabant into the CeA precipitated anxiety-like behavior and hypophagia during palatable food withdrawal (Blasio et al., 2013, 2014a). Rimonabant is associated with an emergence of severe psychiatric side-effects in obese patients (Christensen et al., 2007), which we hypothesize may be due to a precipitation of a withdrawal-like syndrome in a subpopulation of obese individuals abstaining from palatable food as they attempt to lose weight (e.g., by dieting).

## Overeating despite aversive consequences

In drug addiction, loss of control over behavior concerning the drug-seeking or taking in spite of negative consequences represents one of the elements of compulsivity associated with the disorder (Koob and Volkow, 2010; American Psychiatric Association, 2013). Similarly, in many forms of pathological eating, both humans and animals will fail to suppress food seeking and taking in adverse conditions where behaviors would be inhibited (Oswald et al., 2011; Curtis and Davis, 2014; Dore et al., 2014; Velazquez-Sanchez et al., 2014). In humans, negative consequences associated with overeating include social impairment, emotional disturbances, psychiatric disorders, and life-threatening medical conditions associated with weight gain. In animal models, this element of compulsivity is observed as continued consumption of palatable food even when associated with an adverse physical or emotional consequence (e.g., eating while receiving a footshock, or eating in an aversive environment; Figure 1C; Ferragud et al., 2017) (Oswald et al., 2011; Cottone et al., 2012; Curtis and Davis, 2014; Rossetti et al., 2014; Velazquez-Sanchez et al., 2015). This element of continued overeating despite aversive consequences is characterized by failure of inhibitory control processes contributing to a loss of control over eating behavior. Indeed, high trait impulsive action (i.e., inability to withhold a response) has been shown to predict compulsive-like eating behavior (Velazquez-Sanchez et al., 2014).

Inhibitory control over behavior is largely regulated by the prefrontal cortex (PFC), and dysfunctions in cortico-striatal circuitries are thought to underlie compulsive eating behaviors (Volkow et al., 2013). In addictive disorders, it is hypothesized that in one functional domain, PFC areas are hyper-responsive to food cues, resulting in high levels of craving. In a separate functional domain, a general hypo-activation of prefrontal circuits involved in inhibitory control results in impulsivity, incentive salience and reengagement of habit systems via the disinhibition of the basal ganglia and negative emotional states via disinhibition of the stress systems of the amygdala (Koob and Volkow, 2016). The two opposing systems have been conceptualized as a “GO” system (dorsolateral PFC, anterior cingulate cortex, and orbitofrontal cortex), which is sensitized in compulsive eating, and a “STOP” system (ventromedial PFC), which is impaired in compulsive eating (Koob and Volkow, 2016). Abnormalities of the PFC are observed in individuals with BED and some forms of obesity. For instance, decreased baseline activity (Volkow et al., 2008) and enhanced food-cue induced activation (Dimitropoulos et al., 2012) is observed in prefrontal cortical areas of obese individuals; and higher BMI has been linked with diminished PFC activity during regulation of palatable food craving (Giuliani et al., 2014; Silvers et al., 2014). Medial prefrontal dysregulation is associated with deficits in inhibitory control (Batterink et al., 2010; Balodis et al., 2013; Hege et al., 2015) and impaired dietary restraint in individuals with BED (Balodis et al., 2013). Furthermore, lower functional connectivity between dmPFC and amygdalar brain regions was found to be associated with higher disinhibited eating behavior (Dietrich et al., 2016).

Multiple neurotransmitter systems in the PFC are involved in the emergence of compulsive eating behavior, including mu-opioid (MOR), Sigma 1 receptors (Sig_1_R), and Trace Amine-Associated Receptor 1 (TAAR1) (Blasio et al., 2014b; Velazquez-Sanchez et al., 2014; Selleck et al., 2015; Smith et al., 2015; Ferragud et al., 2017). In animal models of compulsive-like eating, limited access to a palatable diet resulted in the altered expression of genes coding for the opioid peptides pro-opiomelanocortin (POMC) and pro-dynorphin (PDyn; increased and decreased, respectively) in the medial PFC; in addition, site specific injection of naltrexone, a non-selective opioid receptor antagonist, into the PFC was able to reduce binge-like eating (Blasio et al., 2014b). In binge eating humans, treatment with a MOR antagonist reduced consumption of palatable food (Ziauddeen et al., 2013), motivation for high calorie food stimuli (Cambridge et al., 2013), and hedonic responses toward a sweet food reward (Ziauddeen et al., 2013). Similarly, in animal studies, the Sig_1_R, a receptor involved in alcohol and drug reinforcement (Sabino et al., 2009, 2017), was upregulated in prefrontal cortical brain regions following limited access to a palatable diet, and peripheral administration of a Sig_1_R antagonist blocked binge and compulsive-like eating (Cottone et al., 2012). TAAR1, a receptor expressed in the striatum and prefrontal cortices is activated by trace amines and has been shown to modulate cortical glutamate and dopaminergic transmission (Leo et al., 2014; Espinoza et al., 2015). Protein expression of TAAR1 is decreased in the medial PFC of compulsive-like, binge-eating rats, and TAAR1 agonism injected into the infralimbic cortex blocked excessive intake of palatable food (Figure 2C; Ferragud et al., 2017). TAAR1 agonism also improved perseverative behavior and impulsivity (Espinoza et al., 2015), thus its effects on compulsive-like eating are likely occurring through restoring a loss of function of the “STOP” system. Prefrontal neurotransmitter systems are thought to influence compulsive behavior through the modulation of glutamatergic signaling in cortico-striatal pathways (Kalivas and Volkow, 2005; Cottone et al., 2012). Indeed, prolonged access to palatable food resulted in dysregulated glutamatergic plasticity of NAc neurons (Brown et al., 2015): accordingly, the uncompetitive antagonist of glutamate N-methyl-D-aspartate glutamate receptors (NMDARs) memantine, which was shown to effectively reduce alcohol and drug reward/reinforcement (Popik et al., 2003; Sabino et al., 2013), reduced binge-like eating when microinfused directly into the NAc (Smith et al., 2015).

## Conclusions

The field of mental health is moving toward a transdiagnostic approach to understanding the neurobiological mechanisms underlying psychiatric disorders. At the National Institutes of Health, a Research Domain Criteria (RDoC) initiative by The National Institute of Mental Health is concentrating efforts into the identification of key domains of function common to multiple disorders (The National Institute of Mental Health, 2013). For example, in addiction disorders, an Addiction Neuroclinical Assessment Framework has been proposed that incorporates the 3 major functional domains derived from the neurocircuitry of addiction: Incentive salience, Negative Emotionality, and Executive function (Kwako et al., 2016). Here, measurement of these domains in epidemiologic, genetic, clinical, and treatment studies are hypothesized to provide ultimately a reconceptualization of the nosology of addiction disorders for better prevention and treatment (Kwako et al., 2016).

Under this perspective, a better understanding of the construct of compulsive eating is warranted. Within the preclinical field, development and use of appropriate animal models that adequately model these functional domains is critical. Modeling complex behavioral constructs, such as those as presented in this review, may lead understanding of the development and progression of underlying neurobiological processes of the elements of compulsive eating behavior. Knowledge of the vulnerability factors, neuroadaptive mechanisms, and their interactions that lead to compulsive eating behavior has the potential to significantly improve behavioral and pharmacological intervention for millions of people.

## Author contributions

All authors made substantial contributions to conception and design of this review. CM drafted the manuscript. VS, GK, and PC substantially and critically revised it for intellectual content. All authors gave final approval for its submission.

### Conflict of interest statement

The authors declare that the research was conducted in the absence of any commercial or financial relationships that could be construed as a potential conflict of interest.
